# Men, Prostitution and the Provider Role: Understanding the Intersections of Economic Exchange, Sex, Crime and Violence in South Africa

**DOI:** 10.1371/journal.pone.0040821

**Published:** 2012-07-20

**Authors:** Rachel Jewkes, Robert Morrell, Yandisa Sikweyiya, Kristin Dunkle, Loveday Penn-Kekana

**Affiliations:** 1 Gender and Health Research Unit, Medical Research Council, Pretoria, South Africa; 2 School of Public Health, University of the Witwatersrand, Johannesburg, South Africa; 3 Research Office, University of Cape Town, Cape Town, South Africa; 4 Rollins School of Public Health, Emory University, Atlanta, Georgia, United States of America; 5 Centre for Health Policy, Faculty of Health Sciences, University of the Witwatersrand, Johannesburg, South Africa; Central Institute of Educational Technology, Canada

## Abstract

**Background:**

South African policy makers are reviewing legislation of prostitution, concerned that criminalisation hampers HIV prevention. They seek to understand the relationship between transactional sex, prostitution, and the nature of the involved men.

**Methods:**

1645 randomly-selected adult South African men participated in a household study, disclosing whether they had sex with a woman in prostitution or had had a provider relationship (or sex), participation in crime and violence and completing psychological measures. These became outcomes in multivariable regression models, where the former were exposure variables.

**Results:**

51% of men had had a provider relationship and expected sex in return, 3% had had sex with a woman in prostitution, 15% men had done both of these and 31% neither. Provider role men, and those who had just had sex with a woman in prostitution, were socially conservative and quite violent. Yet the men who had done both (75% of those having sex with a woman in prostitution) were significantly more misogynist, highly scoring on dimensions of psychopathy, more sexually and physically violent to women, and extensively engaged in crime. They had often bullied at school, suggesting that this instrumental, self-seeking masculinity was manifest in childhood. The men who had not engaged in sex for economic exchange expressed a much less violent, more law abiding and gender equitable masculinity; challenging assumptions about the inevitability of intersections of age, poverty, crime and misogyny.

**Conclusions:**

Provider role relationships (or sex) are normative for low income men, but not having sex with a woman in prostitution. Men who do the latter operate extensively outside the law and their violence poses a substantial threat to women. Those drafting legislation and policy on the sex industry in South Africa need to distinguish between these two groups to avoid criminalising the normal, and consider measures to protect women.

## Introduction

South Africa is reviewing its legislation on the sex industry and prostitution. Public health concerns centre on the vulnerability of women in prostitution to HIV and violence, which are magnified by the criminalisation of buying and selling of sex. Policy drafters perceive a need to incorporate a broader understanding of the arena of economic exchange and sex, as they grapple with definitions of prostitution and transactional sex, questions of what constitutes normative behaviour, and the role of the State in regulating sexual practices and the sex industry. Whilst there has been some local research on the sex industry and health impact of prostitution and transactional sex for women (e.g. [1], [2], [3], [4], [5], [6], much less has been published on the sex industry and men. Whilst perceived as important, the intersections of economic exchange or provision, masculinities, crime, violence and sex have been little explored.

There are different ways of ‘being a man’ in South Africa, but among the largest section of the adult male population – low income Black Africa men - the social ideal positions men as providers, for their girlfriends, wives or families [7]. The provider role commonly features in masculinities globally, but there are differences with the way it is performed. It is an emphasised theme throughout South African relationships and has historically been an essential prerequisite for marriage [7]. Men often give women gifts to demonstrate their sincerity and ability to provide, before they agree to the relationship [8]. Historically men would enter marriage only when able to provide for the establishment of a homestead [7]. With rising joblessness and widespread poverty very many men do not marry and even fail to meet minimal standards of (co-)providing for their children [9]. Yet the provider role continues to frame relationships with women, particularly those whose own economic position is precarious. From the start of dating, women expect men to assume a provider role and men internalize this expectation, elevating it to a key marker of masculinity. Those who fail to provide are considered to be ‘unmanly’. This speaks to the relational construction of gender identities and a widely held belief among men that success with women is predicated on them behaving as women expect (c.f. [7], [8], [10], [11].

There is diversity among men (and within an individual man) in the degree to, and circumstances in, which ‘providing’ is performed, as well as in connections between a provider role and ideas and practices that are socially conservative and gender inequitable. It is possible to perceive the provider role and entailed expectations as positioned on a spectrum, which includes both the highly instrumental position underlying transactional relations with women, which may be expressed as ‘I want women so I give or pay, then I expect to get sex’ as well as a related but softened patriarchal position ‘I provide because I am a man and as a male provider, my women should be sexually available’. A variant of this position could be expressed in yet ‘softer’ terms: ‘I provide because I am a man. My partner/wife, as a woman, gives me sex. We provide for one another’. The latter two views are related to a traditional view of marriage where men provide and expect conjugal rights. South African men’s role as provider and quid pro quo of access to women’s bodies is ritualised in the marriage practice of giving lobola (bride wealth) to the woman’s family (e.g [7], [12], [13]. Many women acknowledge the terms of this exchange, as measured by relatively high (if declining) levels of agreement that a woman married with *lobola* cannot refuse her husband sex [8], [14]. Some men are proud to perceive themselves as having relationships (or sex) predicated on their provision, whilst others resent this expectation of women, particularly in a context where their ability to provide is strained by poverty [15].

Many authors have linked ideas of male sexual entitlement to the objectification of women’s bodies, denial of female sexual agency, to sexual violence and patriarchy [16], [17]. Yet others argue that women’s sexuality is a source of power [18], [19] and in African contexts of poverty, have described how women use sexual power to make their way [20], [21]. Women engaging in transactional sex, whether as girlfriends of ‘sugar daddies’ or meeting their materials needs and wants from their multiple and concurrent partners [4], [22], [23], are often represented, and represent themselves, as breaking the gender mould and forging new roles for women. But there are decided limits to leveraging short-term economic gain from sex, as it perpetuates male ideas of the provider role within a normative matrix of gender and power over women, and reinforces men’s claim to sex as a quid pro quo for being the provider. Thus gender emancipation through sex remains elusive [8], as vividly shown by Leclerc-Madlala’s informants [4].

Qualitative research shows that both women and men distinguish between sex for remuneration (whether referred to as transactional sex or the male provider role with entailed sexual entitlement) and the stigmatised category of ‘prostitution’ [4], [7], [22], [24]. Thus as an emic category, that is one defined in terms meaningful to the actors involved, ‘prostitution’ is viewed as having a clear definition, whereas when viewed as an etic category (defined by external observers) it becomes much less clear how this is distinguished from transactional sex. The proposed definition of ‘prostitution’ developed by the South African Law Reform Commission is “the exchange of any financial or other reward, favour or compensation for the purpose of engaging in a sexual acts” illustrates these difficulties as it clearly subsumes the emically distinct activities of transactional sex/men’s provider role [25] (p.10).

A question emerges about whether men who engage with the two emic categories (provider role with sexual entitlement and having sex with a woman in prostitution) vary from each other or from men who do neither. There has been little research on this question in South Africa and globally, but evidence available suggests some differences. South African young rural men who have had transactional relationships, or sex, are much more likely to have perpetrated all forms of gender based violence than those who have not [26]. North American men who have had sex with a woman in prostitution are more likely to have been sexually violent towards all women [27], [28]. In Scotland, the more often men have frequented women in prostitution, the greater the likelihood of their having been sexually violent towards non-prostitute partners [29]. These findings may be unsurprising, given that the notion of men’s sexual entitlement emerges from a gender order that subordinates women and thus legitimates men’s dominance and control over them [30]. On the other hand, variations among men in the interpretation of the provider role point to the need to interrogate more deeply what might be seen as a causal relationship between being a provider and being violent or misogynistic.

The aim of this paper is to deepen understanding of men and masculinities as they relate to economic exchange for sex and gender relations through exploring four indicator positions. The first is the provider relationship (or sex), the second is having sex with a woman in prostitution, the third is having had both of these, and the fourth of having had neither. This last category may include men who ‘provide’ but do not view their sexual relations as having been predicated on this. We describe associations between these four indicator positions and the prevalence of practices of gender relations, engagement with crime and violence and associated psychological attributes, and reflect on how these correlations help us understand the origins and ongoing occurrence of these practices of men. The data presented are from a survey of men and masculinity conducted with a randomly selected sample of South Africa adult men, and thus these men are a random sample of providers and clients of women in prostitution. This paper is the second of two presenting analyses on economic exchange for sex from the dataset. The first paper is published elsewhere [24].

## Methods

Ethics approval was given by the Medical Research Council’s Ethics Committee. The study was undertaken in three adjoining districts in the Eastern Cape and KwaZulu-Natal provinces. The area spans traditional rural land, commercial farms, small towns, villages, and a city. Its population includes all South Africa’s racial groups, several ethnic groups (predominantly Xhosa and Zulu) and socio-economic backgrounds. The sample used a two stage proportionate stratified design to identify a representative sample of men living in households. Using the 2001 census as the primary sampling frame, 222 census enumeration areas (EAs) were selected as the primary sampling unit, stratified by district, and with numbers proportionate to district population size. The sample was drawn by Statistics South Africa. Households in each EA were mapped and twenty were systematically selected. In each household one eligible man was randomly selected to take part in the interview. Eligible men were aged 18–49 years and had slept there the night before.

Of the 222 selected EAs, two (0.9%) had no homes, and we could not interview in five (2.3%) because permission from the local political gatekeepers was declined (1) or we could not identify an eligible household after multiple visits at different times of day (4). In all of these, we found many selected households lacked an eligible man. We did not use replacement. We completed interviews in 215 of 220 eligible EAs (97.7%). We sampled a total of 4473 visiting points. Of these, 1353 (37.1%) were found to contain no eligible man, 2298 (51.4%) contained at least 1 eligible man, and 822 (18.4%) could not be rostered for eligibility after a minimum of 3 attempts at contact. We completed interviews in 1737 of 2298 (75.6%) enumerated and eligible households.

Interviews took 45–60 minutes, and were conducted in isiXhosa or isiZulu or English with data collected using self-completion on APDAs (Audio-enhanced Personal Digital Assistants). Research participants were approached by members of the study-trained, male fieldwork team and invited for interview. A fieldworker stayed on hand through out the interview in case participants had problems or wished clarification or to raise concerns.

### Measurement

Categorical variables measured age, education, race, marital status, employment, and income. Men were asked, separately for main partners and on-going secondary partners (*makhwapheni* (pl.) an indigenous term in Nguni languages), “Do you think any of them become involved with you because they expected you to do, or because you did do any of the following:” with yes/no response options for providing: food, clothes, cell phone or transportation; school fees or residence fees; somewhere to stay; cosmetics; items for children or family; handyman work; cash or money to pay bills; and anything else that she could not afford. For once-off partners, men were asked a very similar question with response options for food, clothes, or cosmetics; transportation; a place to sleep for the night; handyman work; cash or money to cover expenses; and anything else that she could not afford. Responding affirmatively to any item for any partner rendered a man to have engaged in a provider relationship (or sex). We do not use the term ‘transactional sex’ because we acknowledge that there would have also been emotional and social dimensions to men’s provider relationships. Further this measure was of men’s perceptions of women’s motives and we recognise that perceptions of motivation may differ between parties in a sexual encounter. We also asked men “Have you ever had sex with a prostitute?” In order to understand the overlap between these a four level variable was derived (categories described above).

Three gender attitudes scales were used, Gender Equitable Men (GEM) scale (10 items) [31] (Cronbach’s alpha 0.78); the Hostility Towards Women scale (5 items) (Cronbach’s alpha 0.77); and the Rape Myths scale (4-items) (Cronbach’s alpha 0.76) [32].

Three acts of gender based violence were measured: lifetime perpetration of physical intimate partner violence (IPV); rape of women partners and non-partners; and perpetration of male rape. Five items asked about acts of IPV ranging from slapping to use of a weapon (after [33]. The variable analysed was of two or more acts of violence, versus one or none. Seven questions asked about rape perpetration, framed around sex with women ‘without consent’ or ‘forced’ [34], [35]. Two questions asked about rape of a man or boy.

Bullying at school was measured on an 8 item scale (Cronbach’s alpha 0.76). These questions were developed for the study and a typical item was “When a girl thought she was smart at school we would put her in her place by using her sexually”. We asked about frequency of engagement in stealing or robbery (8 items) (modified from Tremblay et al [36]) (Cronbach’s alpha 0.81). Men were asked about current weapon ownership, ever having possessed an unlicensed gun, gang membership and how often in the last year they had smoked dagga (cannabis).

The 4 item empathy scale was adapted from Abbey et al [37](Cronbach’s alpha 0.80). We used part of a standard instrument, the Psychopathic Personality Inventory Revised (PPI-R), developed in the United States to measure dimensions of psychopathy. We could not externally validate it for the South African population, but did test item validity during cognitive interviews. Blame externalisation and Machiavellian egocentricity are two core affective and interpersonal deficits of psychopathy [38]. Blame externalisation is a perception of the world as hostile and others being at fault for one’s problems and Machiavellian egocentricity is a measure of narcissism and ruthless attitudes towards others [38]. The PPI-R has considerable item duplication to enable internal reliability to be assessed. We shortened the measures, excluding duplicates, and used 6 of 15 items for blame externalisation and 7 of 20 items for Machiavellian egocentricity. Our Cronbach’s alphas were 0.82 and 0.74 respectively. A typical measure of blame externalisation is “I get blamed for many things that aren’t my fault”, whereas of Machiavellian egocentricity is “I quickly get annoyed with people who do not give me what I want”. We dichotomised the scales and present the proportion scoring in the upper third of the scale versus the lower two-thirds. For blame externalisation 28.4% were in the upper third and for Machiavellian egocentricity 18.5% were in the upper third. These were adapted and reproduced by special permission of the Publisher Psychological Assessment Resources, Inc., 16204 North Florida Avenue, Lutz, Florida 33549, from the Psychopathic Personality Inventory- Revised by Scott O. Lilienfield, Ph.D., Copyright 2005 by PAR, Inc.

### Ethical Issues

The men were informed about the study, given an information sheet and signed consent. They received R25 (US $3.2) for the interview and those giving blood sample for an HIV test were given a further R25 (data not presented). Since the questionnaire asked men to disclose a range of criminal acts and South African law does not privilege research data, interviews were conducted anonymously. No identifying details of the men or their households were kept after the interview and the consent forms could not be linked.

### Data Analysis

The study design provided a self-weighted sample of households. Data files were collated and analyses were carried out using Stata 10.0. All procedures took into account the two stage structure of the dataset, with stratification by district and the EAs as clusters. The main exposure variable of interest in this analysis was the four level provider role/paid sex derived variable. For Table 1 the distributions of categorical variables by provider role/paid sex category were summarised as percentages. Pearson’s chi was used to test associations between categorical variables. The analyses were based on 1645 men who had no missing data for classification on the four level commodified sex variable. No efforts were made to replace missing data.

**Table 1:** Social and demographic characteristics of men who have had a relationship or sex predicated on their material provision, sex with a woman in prostitution or both of these or neither.

	None (N = 510) %	Relationship or sex predicated on men providing(N = 839) %	Sex with a woman in prostitution(N = 51) %	Both(N = 245) %	p value
**Age:** 18–24	62.2	48.3	45.1	46.1	<0.0001
25–34	21.4	34.2	33.3	38.8	
35–49	16.5	17.5	21.6	15.1	
**Education:** no morethan primary	15.8	22.8	17.7	12.7	0.0003
secondary	43.4	39.6	43.1	40.0	
Matric or higher	40.8	37.6	39.2	47.4	
**Race:** African	84.5	88.8	62.8	78.8	<0.0001
Coloured	2.4	3.8	9.8	8.6	
Indian	10.8	6.6	25.5	10.2	
White	2.4	0.8	2.0	2.5	
**Worked in the last year**	50.6	54.5	60.8	69.4	<0.0001
**Monthly income:** none	57.9	48.6	42.6	32.1	<0.0001
R 1–500	15.7	19.7	6.4	15.8	
R 500–2000	14.2	21.5	21.3	32.9	
R 2001–5000	6.5	6.4	21.3	10.7	
R 5000+	5.8	3.9	8.5	8.6	
**Marital status:** married	25.7	22.7	34.7	16.5	<0.0001
cohabiting	6.7	11.7	10.2	19.9	
divorced/widowed	2.1	4.3	4.1	3.8	
single	65.5	61.4	51.0	59.8	

To measure associations between psychological measures and behavioural outcomes and the provider role/paid sex variable, we used multiple regression or logistic regression as appropriate depending on the outcome variable. For these models the reference category for the variable was taken as the most highly prevalent (and thus currently normative) category, having had a relationship or sex predicated on men providing. Because of clustering of men within EAs we present random effects multi-variable regression models. For each outcome of interest we included the four level provider role/paid sex exposure variable as well as age, race, income, marital status, education and a term for stratum. All variables were retained.

## Results

The men in the study were aged 18–49 years, with most (70%) under 30. Fewer than half had completed high school. They came from all racial groups, but 86% were Black African. Only half of the men worked, mostly earning little (under R2000 ($300) per month). Most had never married, and only 27% were currently married. The largest group of men (n = 839 51%) had had a provider relationship, a further 51 (3%) men had had sex with a woman in prostitution but not a provider relationship, 245 (15%) men had had both and 510 (31%) had had neither.

The provider relationship men were most likely to be African and never married (Table 1). Men who had just had sex with a woman in prostitution were much more likely to be Indian and Coloured, had the highest income and were most likely to be married. Those who had had both were most likely to have completed school, to work and more were cohabiting. Those who had done neither were younger, mostly African, and more often single and unemployed.

The prevalence of disclosed engagement in bullying at school and gender-based violence among the groups of men is presented in Table 2. There was no difference in mean score for bullying at school between men who had had a provider relationship and those who had just had sex with a woman in prostitution, but having had both was associated with a significantly elevated risk of bullying. Men who had had neither were at significantly less likely to have done so.

**Table 2:** Prevalence and adjusted odds ratios or coefficients from multiple variable regression models for associations between having had a relationship or sex predicated on their material provision, sex with a woman in prostitution or both of these or neither.*

	Sexual bullying at school score				Rape of a woman				Rape of a man			
	mean	Coef**	95% CI	p value	%	aOR***	95% CI	p value	%	aOR***	95% CI	p value
Relationship or sex predicated on men providing (n = 839)	10.68	ref			28.0	1.00			2.9	1.00		
Sex with a woman in prostitution (n = 51)	11.37	0.75	−0.14, 1.65	0.102	29.4	0.93	0.46, 1.88	0.843	0	–		
Both (n = 245)	11.80	1.18	0.73, 1.63	<0.0001	53.5	2.65	1.91, 3.67	<0.0001	8.2	2.89	1.36, 6.14	0.006
Neither (n = 510)	9.64	−1.02	−1.38, −0.67	<0.0001	12.6	0.37	0.26, 0.51	<0.0001	1.0	0.32	0.10, 0.99	0.047
	**>1 episode of physical intimate partner violence**											
	**%**	**aOR** ***	**95% CI**	**p value**								
Relationship or sex predicated on men providing (n = 839)	34.8	1.00										
Sex with a woman in prostitution (n = 51)	40.0	1.23	0.65, 2.35	0.521								
Both (n = 245)	57.1	2.3	1.68, 3.17	<0.0001								
Neither (n = 510)	14.4	0.35	0.25, 0.48	<0.0001								

* each model adjusted for age, race, education, income, marital status, stratum.

** Coefficient from multiple regression model.

*** adjusted odds ratio from logistic regression model.

There were significant differences among the groups in rape perpetration. The prevalence of having raped a woman was very similar for men who had had a provider relationship and those who had just had sex with a woman in prostitution, and was more than twice that found among men who had had neither. More than half (54%) of the men who had done both had raped (Table 2). The latter group were also significantly more likely than provider relationship men to have raped a man, with 1 in 13 (8%) having done this. Only 1% of those men who had done neither had raped a man.

The prevalence of being physically violent towards a woman partner on multiple occasions also rose substantially across the provider role/exchange categories, from 14% among those who had had neither to 57% among those having had both (Table 2). Men who had just had a provider relationship and those who had just had sex with a woman in prostitution differed little in their likelihood of having been violent on multiple occasions (about a third had done this), in comparison those who had done both had more than twice the odds, and those who had done neither, had a 65% lower odds.

Table 3 presents associations between the four masculine positions and a range of measures of weapon possession and engagement with illegal activities. One third of the men who had been in a provider relationship or just had sex with a woman in prostitution had a weapon, compared to nearly two-thirds of men (59%) who had done both and 16% of men who had done neither. The adjusted odds ratios indicate that men who had done both were more than three times as likely to have a weapon than men who had had a provider relationship, and that those who had had neither were 64% less likely.

**Table 3:** Prevalence and adjusted odds ratios from logistic regression models for associations between having had a relationship or sex predicated on their material provision, sex with a woman in prostitution or both of these or neither and weapon possession, gang membership, drug use and property crime.*

	Has a weapon (gun or other)				Ever had an illegal gun				Ever a gang member				
	%	aOR	95% CI	p value	%	aOR	95% CI	p value	%	aOR		95% CI	p value
Relationship or sex predicated on men providing (n = 839)	31.0	1.00			10.5	1.00			8.4	1.00			
Sex with a woman in prostitution (n = 51)	38.0	1.27	0.65, 2.50	0.484	20.0	1.66	0.72, 3.81	0.232	24.0	2.69		1.18, 6.11	0.018
Both (n = 245)	58.7	3.28	2.33, 4.60	<0.0001	26.9	2.89	1.91, 4.39	<0.0001	28.5	4.01		2.57, 6.25	<0.0001
Neither (n = 510)	16.4	0.36	0.26, 0.49	<0.0001	3.4	0.28	0.15, 0.50	<0.0001	5.6	0.56		0.34, 0.93	0.026
	**Last year illicit drug use**				**Involvement in theft or robbery**								
					**never**	**1–2 times**	**3+ times**						
	**%**	**aOR**	**95% CI**	**p value**	**%**	**%**	**%**	**aOR** **	**95% CI**		**p value**		
Relationship or sex predicated on men providing (n = 839)	37.4	1.00			43.9	26.8	29.3	1.00					
Sex with a woman in prostitution (n = 51)	64.0	3.06	1.52, 6.19	0.002	22.5	24.5	53.1	2.43	1.20, 4.89		0.013		
Both (n = 245)	61.9	2.54	1.81, 3.55	<0.0001	14.2	21.3	64.6	3.57	2.52, 5.05		<0.0001		
Neither (n = 510)	26.1	0.51	0.39, 0.69	<0.0001	57.3	19.2	23.6	0.69	0.51, 0.93		0.016		

* each model adjusted for age, race, education, income, marital status, stratum.

** model of factors associated with having done theft or robbery 3+ times.

Ever having had an illegal gun was reported by many of the men, with between a third and a half of currently weapon-owning men having ever had one. One in five men (20%) who had ever had sex with a woman in prostitution and one in four men in the ‘both’ group had had an illegal gun (27%). The adjusted odds ratios show no difference between the men who had had a provider relationship and those who had sex with a woman in prostitution, but those who had had both were nearly three times as likely to have had an illegal gun. Very few of those who had had neither had one (3%) (72% lower odds).

Having been a gang member was strongly associated with having had sex with a woman in prostitution. A quarter of all men who had sex with a woman in prostitution had been in gangs. This contrasted with 8% of men who had a provider relationship and 6% among men who had had neither. The adjusted odds ratios show a more than 2 fold increased likelihood of gang membership among those who had just had sex with a woman in prostitution (aOR 2.69), rising to a four fold increased likelihood among those who had also had a provider relationship (aOR 4.10). Men who had done neither had nearly half the likelihood of having been in a gang (aOR 0.56).

Use of illicit drugs in the previous year followed the same pattern. 60% of drug-using men had had sex with a woman in prostitution. This compared to just over a third of those who had a provider relationship (37%) and a quarter of those who had had neither (26%). This pattern was confirmed by the adjusted odds ratios (Table 3).

The same pattern was also seen for men’s stealing and robbery. Many of the men interviewed disclosed multiple episodes of theft and robbery, with 29% of men who had had a provider relationship had been involved on three or more occasions. There was however, a very strong association with engagement in sex with a woman in prostitution, with over half of the men who had had this having been involved three or more times in theft or robbery. The adjusted odds ratios for having sex with a woman in prostitution alone was aOR 2.43 and for the ‘both’ group was aOR 3.57. Men who had had neither were significantly less likely (aOR 0.69).

Table 4 presents analyses examining a range of psychological variables. Across all of these the men who had only a provider relationship and those who had only had sex with a woman in prostitution were very similar. In contrast, the men who had had both scored significantly more gender inequitable on the three scales, although they did not differ on empathy. When examining the scales measuring the two psychopathic traits blame externalisation and Machiavellian egocentricity, the men who had had both were significantly more likely to be in the upper two-thirds of the scale, with the adjusted odds ratios elevated by 50% and 130% respectively.

**Table 4:** Prevalence and adjusted odds ratios or coefficients from multiple variable regression models for associations between having had a relationship or sex predicated on their material provision, sex with a woman in prostitution or both of these or neither and psychological measures.*

	Gender equitable men scale				Rape myth scale				Hostility towards women scale			
	mean	Coef	95% CI	p value	mean	Coef	95% CI	p value	mean	Coef	95% CI	p value
Relationship or sex predicated on men providing (n = 839)	23.00	ref			9.71	ref			4.48	ref		
Sex with a woman in prostitution (n = 51)	23.20	−0.44	−2.08, 1.21	0.60	9.21	0.39	−0.53, 1.31	0.41	3.80	−0.10	−1.09, 0.90	0.85
Both (n = 245)	22.45	−0.99	−1.80, −0.17	0.02	9.89	0.59	0.14, 1.04	0.01	4.34	0.49	−0.01, 0.98	0.06
Neither (n = 510)	24.49	1.40	0.75, 2.04	<0.0001	9.12	−0.59	−0.94,−0.24	<0.0001	3.31	−1.14	−1.54, −0.75	<0.0001
	**Psychopathy: blame externalisation**				**Empathy**				**Psychopathy: Machiavellian egocentricity**			
	**%**	**aOR**	**95% CI**	**p value**	**mean**	**Coef**	**95% CI**	**p value**	**%**	**aOR**	**95% CI**	**p value**
Relationship or sex predicated on men providing (n = 839)	40.6	ref			13.36	ref			20.0	ref		
Sex with a woman in prostitution (n = 51)	39.6	1.03	0.54, 1.97	0.92	15.78	1.25	−0.46,3.97	0.15	14.6	0.92	0.39, 2.19	0.858
Both (n = 245)	51.1	1.50	1.08, 2.07	0.014	13.61	−0.32	−1.15, 0.52	0.46	30.2	2.31	1.59, 3.34	<0.0001
Neither (n = 510)	22.8	0.41	0.31, 0.54	<0.0001	14.94	1.91	1.26, 2.56	<0.0001	10.6	0.46	0.32, 0.67	<0.0001

* each was modelled separately using multiple regression and each model adjusted for age, race, education, income, marital status, stratum.

The men who had done neither of these scored quite differently from the men who had a provider relationship. They were more gender equitable, scoring very much higher on the GEM scale, were much less likely to believe rape myths, much less hostile towards women and demonstrated significantly higher empathy. A very much smaller proportion scored in the upper two-thirds of the two scales measuring psychopathic traits (adjusted ORs of 0.41 and 0.46 for blame externalisation and Machiavellian egocentricity).

## Discussion

In total 66% of men, from a range of social groups, had had a provider relationship (or sex), i.e. they embraced the hegemonic masculine ideal that as men they should ‘provide’ for women and translated this instrumentally into an entitlement to sex. Just under a quarter of men in a provider relationship (15% of all men) had also paid for sex with a woman in prostitution i.e. the men who had done ‘both’. They constituted the majority of men who had had sex with a woman in prostitution, but not all of them, as a quarter of this group had not had a provider relationship. Although having a provider relationship was normative, not all men had had one. A third of men (31%) had neither had sex with a woman in prostitution, nor a provider relationship (even if they did perhaps see themselves as providers).

These groups of men differed in age, education, race, income and marital status. The men who had done ‘both’ were relatively more advantaged than the others. They were better educated, more were Coloured or Indian, employed and earning more (if still mostly very low income). They were the most patriarchal or misogynistic in their attitudes, being more gender inequitable, hostile towards women, and expressive of rape myths. Yet all men who had having ‘provider relationships’ were socially conservative. They scored significantly less gender equitable on attitudes scales when compared to the men who neither had a provider relationship nor had had sex with a woman in prostitution. The men who had provider relationships and those who had only had sex with a woman in prostitution did not differ on gender equity measures. Our findings are in keeping with international literature, which generally notes the hostility towards women of men who have sex with women in prostitution [29].

The other psychological measures and gender-based violence perpetration showed a similar pattern. The men who had had ‘both’ scored the highest on psychopathic traits and a very high proportion had raped women (and comparatively speaking also men), and had been physically violent on multiple occasions towards women. The men who had had a provider relationship or just had sex with a woman in prostitution were very similar, and held a middle ground, whilst those who had done neither were significantly lower scoring on psychopathy and much less likely to have used gender-based violence. They were also significantly higher scoring on empathy.

The analysis of the intersection of the groups of men and weapon ownership and participation in crime again showed a strikingly high proportion of men in the ‘both’ group who had a weapon, an illegal gun, been in a gang, used drugs and had been involved on multiple occasions in theft and robbery. These men were 2.5–4 times more likely to have been violently, or criminally, engaged, than the men who just had a provider relationship. Again there was a most striking contrast with the men who had had neither, they were 5 times less likely to have done many of these practices than men who were in the ‘both’ group.

Weapon ownership and participation in crime were the only areas where the men who had just had sex with a woman in prostitution differed from the men who had just had a provider relationship. When prevalences and confidence intervals of effect measures were examined there was no apparent difference between the men who had just had sex with a woman in prostitution and those who had done this in the ‘both’ group. The association between the sex industry and crime and drugs is well described (e.g. Leggett [39]), but there has been less research on clients in the industry, as opposed to those men and women who work in or run it (exceptions include Monto & McRee [27]). There may have been overlap in our sample between men who profit from the industry and male clients, but we did not ask about this. A likely conclusion is that men who are unperturbed by the illegality and stigma associated with buying sex with a woman in prostitution [7] are more likely to have parted from other social mores and thus more willing to use drugs, have a history of engagement in anti-social practices of gang membership and robbery.

The measures of psychopathic traits (blame externalisation and Machiavellian egocentricity) point to psychological processes which enable perpetration of violence, engagement with crime and instrumentality in relations with women. We used two of eight sub-scales of the PPI-R, shortened them and they had not been standardised for the South African population as a result they can not be interpreted as giving diagnoses of psychopathy [38]. Nonetheless in the United States a score in the upper most third of the scale is seen as most likely to be indicative of clinically diagnosed psychopathy, and so we have used this as the cut point in this analysis. The proportions in the uppermost third were very high in this study. It is easy to imagine why Black South Africans may score highly (and not necessarily irrationally) on blame externalisation, given the country’s history, whilst at the same time noting the marked patterning of this psychological measure with different masculine positions. The very high scores on Machiavellian egocentricity point to a psychological position that allows for the perpetration of violence. These high scores are very likely to reflect very high levels of exposure to trauma in childhood [40], [41].

In this paper we have sought insights into South African men who position themselves as having provider relations with women, and through this feel entitled to sex, and the related practice of having sex with a woman in prostitution. We have shown that when these positions are taken as mutually exclusive, the men who adopt them are extremely similar. These are socially conservative positions, where men endorse patriarchal relations with women and many of them have engaged in acts of gender-based violence. When these categories overlap, they become indicators for a group of men who have highly instrumental relationships towards women, and as the crime indicators suggest, quite probably, if in different ways, towards other men. Thus the notion of men as providers, whilst culturally normative, spans a range of men’s positions, some of which are far from acceptable male behaviour.

The men who have had sex with a woman in prostitution show diversity, a minority are men who in their attitudes and behaviour would be viewed as ‘normal’ South African men. The majority (75%) are a very violent group of men. Our analysis makes it is easy to understand why women in prostitution, whether in South Africa or internationally, so often experience violence from their male clients [42], [43], [44], [45], [46].

The question of whether the experience of buying sex enhances men’s sense of sexual entitlement, promotes sexual preoccupation and thus heightens likelihood of raping and other violence towards other women is of great concern to prostitution policy makers [25]. This is critical for consideration during review of legislation on the sex industry, because decriminalisation (or partial legalisation) of prostitution and regulation have invariably resulted in at least short term expansion of the sex industry [47]. It is not possible to answer this from a cross-sectional study, however school bullying is very likely to have preceded men’s acts of sex with a woman in prostitution and probably provider relationships. It is thus interesting that the men who had had ‘both’ had a significantly higher score on the school bullying and that the men who had had ‘neither’ scored significantly lower. This at least raises the possibility that the psychological characteristics and ideational framework of dominance that enables men to later define their relations with women in conservative gender terms or frankly instrumentally, is constructed at a relatively early age, and certainly during adolescence, if not earlier in childhood. This would be supported by reference to literature in developmental psychopathology [41]. The levels of crime and violence disclosed by most men who had sex with a woman in prostitution suggest a preference for an unregulated world. The decriminalisation of sex work, therefore, would be unlikely to make much difference for this particular group of (dangerous) men.

The male positions on economic exchange and sex that have been used to frame this paper categorise men on the basis of their having *ever* had sex on particular terms (as defined above). We do not know how often they have done this, or how the men most commonly frame their sexual relations. Yet we have shown that these positions are correlated with a range of psychological attributes, ideas and practices of gender relations and social relations more broadly. The study was cross-sectional and it is impossible to know the temporal sequence of most variables, yet the analysis is of interest because the psychological attributes or gender antecedents of all behaviours measured are likely to have been established considerably earlier than the behaviours were enacted or psychological variables measured. A strength of the study was the use of APDAs for data collection, as these provided a confidential environment in which disclosure of anti-social and illegal behaviour could be enabled. Through removing the face to face component to interviews, APDAs greatly reduced the performative aspect of interview responses and so gave us more confidence that there would not have been a problem of over-reporting. Under-reporting is a potential problem in research on crime and violence. It is impossible to estimate the magnitude of this in the study. Recall bias is a potential problem with any study of this nature. Self-completion of the questionnaire and the option of skipping questions resulted in some missing data on some items. We have not replaced missing values. We did not retain information on the number of eligible men per household and so were not able to weight the analysis for this, but we have no reason to believe this would have made much difference to the estimates of association [48].

### Conclusions

This study has shown that for men to occupy a provider role is a normal, normative behaviour that meshes with women’s expectations and ideas of reciprocity The provider role is often (but not always) associated with a notion of sexual entitlement. This behaviour is part of a conservative, provider-orientated masculinity that is hegemonic in South Africa. This contrasts with having sex with women in prostitution, which is a practice of a minority of men. A challenge for South African law makers is to develop a definition of ‘prostitution’ which does not subsume the normative emic category of having provider role relationships or sex. The study has also shown that most men who have had sex with women in prostitution are not ‘normal’ men. They are all more likely to have engaged in a range of illegal practices. Further the largest group of these men, those who had also had sex or relationships predicated on their occupying a provider role, displayed a self-focused, instrumental masculinity. They had the most pronounced gender inequitable attitudes and psychological attributes indicating ruthlessness in interpersonal relations. They were also much more likely to have engaged in a range of acts of gender-based violence. As such they pose a considerable threat to women in prostitution.

Our research has also shown that there is another minority masculine position that is more considerate and gender equitable, where men have neither paid for sex nor perceived sexual entitlement flowing from a role as providers. The ideas and practices of men are constitutive of the three masculine positions described in this study, but also flow from them. These positions are historically constructed though the teenage years (as we have no information before) are critical, especially in cases of school bullying. Younger men numerically dominate the more gender equitable position and this opens the possibility of men changing and becoming more gender equitable, but suggest that the interventions needed for this should be focused on boys in childhood. Perhaps they also harbour a glimmer of hope for change among the youngest generation of South African men.

## References

[1] Dunkle KL, Jewkes RK, Brown HC, Gray GE, McIntryre JA (2004). Transactional sex among women in Soweto, South Africa: prevalence, risk factors and association with HIV infection.. Soc Sci Med.

[2] Gould C, Fick N (2008). Selling sex in Cape Town: Sex work and human trafficking in a South African city.. Pretoria/Tshwane: Institute for Security Studies.

[3] Trotter H (2007). Sugar Girls and Seamen A journey into the world of dockside prostitution in South Africa..

[4] Leclerc-Madlala S (2003). Transactional sex and the pursuit of modernity.. Social Dynamics.

[5] Hunter M (2002). The materiality of everyday sex: thinking beyond ‘prostitution’.. African Studies.

[6] Wojcicki J (2002). “She drank his money”: survival sex and the problem of violence in taverns in Gauteng province, South Africa.. Medical Anthropology Quarterly.

[7] Hunter M (2010). Love in the Time of AIDS.. Inequality, gender and Right in South Africa Pietermaritzburg: University of KwaZulu-Natal Press.

[8] Jewkes R, Morrell R (2011). Sexuality and the limits of agency among South African teenage women: theorising femininities and their connections to HIV risk practices.. Social Science & Medicine.

[9] Richter L, Morrell R, editors (2006). *Baba: Fathers and Fatherhood in South Africa*.. Cape Town: HSRC Press.

[10] Lileaas U-B (2007). Masculinities, sport, and emotions.. Men and Masculinities.

[11] Talbot K, Quayle M (2010). The Perils of Being a Nice Guy: Contextual Variation in Five Young Women’s Constructions of Acceptable Hegemonic and Alternative Masculinities.. Men and Masculinities.

[12] Guy J, Walker C (1990). Gender oppression in southern Africa’s precapitalist societies..

[13] Leclerc-Madlala S (2008). Age-disparate and intergenerational sex in southern Africa: the dynamics of hypervulnerability.. AIDS.

[14] Machisa M, Jewkes R, Lowe-Morna C, Rama K (2011). The war at home.. Johannesburg: GenderLinks.

[15] Meth P (2009). Marginalised men’s emotions: Politics and place.. Geoforum.

[16] McKinnon C (1979). Sexual Harassment of Women Workers.. New Haven: Yale University Press.

[17] Russell DE, Bolen R, Bolen M (2000). The epidemic of rape and child sexual abuse in the United States. Thousand Oaks, Ca.. : Sage Publications.

[18] Paglia C (1994). Vamps and Tramps: New Essays.. New York: Random.

[19] Bright S (1997). Sexual State of the Union.. New York: Touchstone Books.

[20] Haram L, Arnfred S (2004). ‘Prostitutes’ or Modern Women? Negotiating respectability in Northern Tanzania..

[21] Silberschmidt M, Rasch V (2001). Adolescent Girls, Illegal Abortions and ‘Sugar-Daddies’ in Dar es Salaam : Vulnerable Victims and Active Social Agents.. Social Science & Medicine.

[22] Wamoyi J, Wight D, Plummer M, Mshana G, Ross D (2010). Transactional sex among young people in in rural northern Tanzania: an ethnography of Young women’s motivations and negotiation.. Reproductive Health.

[23] Nkosana J, Rosenthal D (2007). The dynamics of intergenerational sexual relationships: the experience of schoolgirls in Botswana.. Sexual Health.

[24] Jewkes R, Morrell R, Sikweyiya Y, Dunkle K, Penn-Kekana L (2012). Transactional relationships and sex with a woman in prostitution: prevalence and patterns in a representative sample of South African men BMC Public Health.

[25] South African Law Reform Commission (2009). Discussion paper 0001/2009 Sexual Offences, Adult Prostitution.. Pretoria: South African Law Reform Commission.

[26] Dunkle K, Jewkes R, Nduna M, Jama PN, Levin JB (2007). Transactional sex and economic exchange with partners among young South African men in the rural Eastern Cape: prevalence, predictors, and associations with gender-based violence.. Social Science & Medicine.

[27] Monto MA, McRee N (2005). A Comparison of the Male Customers of Female Street Prostitutes With National Samples of Men.. International Journal of Offender Therapy and Comparative Criminology.

[28] Busch NB, Bell H, Hotaling N, Monto M (2002). Male Customers of Prostituted Women: Exploring Perceptions of Entitlement to power and Control and Implications for Violent Behavior toward Women.. Violence Against Women.

[29] Macleod J, Farley M, Anderson L, Golding J (2008). Challenging Men’s Demand for Prostitution in Scotland: A research report based on interviews with 110 men who bought women in prostitution.. Glasgow: Women’s Support Project & Prostitution Research & Education.

[30] Connell R (1987). Gender and power: Society, the Person and Sexual Politics. Palo Alta, Calif.. : University of California Press.

[31] Pulerwitz J, Barker G (2008). Measuring attitudes toward gender norms among young men in Brazil: development and psychometric evaluation of the GEM scale.. Men and Masculinities.

[32] Burt M (1980). Cultural myths and support for rape.. Journal of Personality and Social Psychology.

[33] Garcia-Moreno C, Hansen HA, Ellsberg M, Heise L, Watts C (2005). WHO Multi-country Study on Women’s Health and Domestic Violence Against Women.. Geneva: World Health Organisation.

[34] Jewkes R, Sikweyiya Y, Morrell R, Dunkle K (2011). Gender inequitable masculinity and sexual entitlement in rape perpetration South Africa: findings of a cross-sectional study PloS One 6..

[35] Sikweyiya Y, Jewkes R, Morrell R (2007). Talking about Rape: Men’s responses to questions about rape in a research environment in South Africa.. Agenda.

[36] Tremblay R, Pagani-Kurtz L, Massc L, Vitaro F, Pihl R (1995). A bi-model prevention intervention for disruptive kindergarten boys: its impact through mid-adolescence.. Journal of Consulting and Clinical Psychology.

[37] Abbey A, Parkhill MR, BeShears R, Clinton-Sherrod AM, Zawacki T (2006). Cross-Sectional predictors of sexual assault perpetration in a community sample of single African American and Caucasian Men.. Aggressive Behaviour.

[38] Lilienfield S (2005). Psychopathic Personality Inventory- Revised Lutz, Florida Psychological Assessment Resources, Inc..

[39] Leggett T (2001). Rainbow Vice.. The drugs and sex industries in the new South Africa London & New York/Cape Town: Zed Books, David Philip.

[40] Mathews S, Jewkes R, Abrahams N (2011). ‘I had a hard life’’: Exploring childhood adversity in the shaping of masculinities among men who killed an intimate partner in South Africa.. British Journal of Criminology.

[41] Fonagy P, Target M (2003). Psychoanalytic Theories: Perspectives from Developmental Psychopathology.. New York: Routledge.

[42] Pauw I, Brener L (1997). Naming the dangers of working on the street.. Agenda.

[43] Karim QA, Karim SS, Soldan K, Zondi M (1995). Reducing the risk of HIV infection among South African sex workers: socioeconomic barriers and gender barriers.. American Journal of Public Health.

[44] Marcus T, Oellerman K, Levin N (1995). Aids and the highways: sex workers and truck driver in KwaZulu-Natal.. Indicator SA.

[45] Church S, Henderson M, Barnard M, Hart G (2001). Violence by Clients towards Female Prostitutes in Different Work Settings: Questionnaire Survey.. British Medical Journal.

[46] Farley M, Barkan H (1998). Prostitution, Violence Against Women, and Posttraumatic Stress Disorder.. Women & Health.

[47] Kelly L, Coy M, Davenport R (2009). Shifting sands: a comparison of prostitution regimes across nine countries.. London: London Metropolitan University.

[48] Korn EL, Graubard B (1995). Examples of differing weighted and unweighted estimates from a sample survey.. American Statistician.

